# Supplementary material for: Anticancer Activity of Natural and Synthetic Chalcones
Source: Int J Mol Sci. 2021 Oct 20;22(21):11306. doi: 10.3390/ijms222111306 (PMC8582663; doi:10.3390/ijms222111306)
Supplement: Supplementary file 1 [file ijms-22-11306-s001.zip › Table S1 structures.pdf]

Table S1. Chemical structures of natural and synthetic chalcones with anticancer activity.

	Structure	Chalcone derivative
1	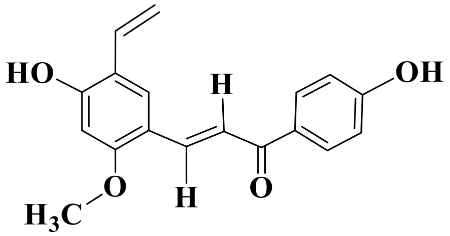	Licochalcone A
2	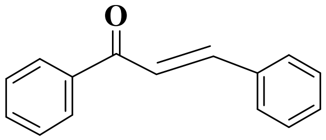	trans-chalcone
3	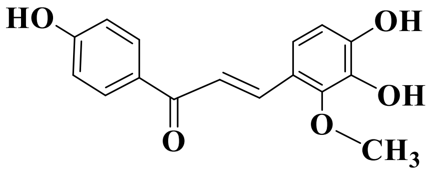	Licochalcone B
4	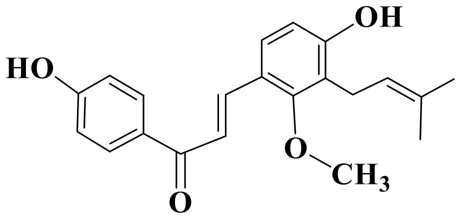	Licochalcone C
5	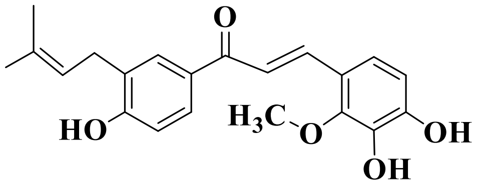	Licochalcone D
6	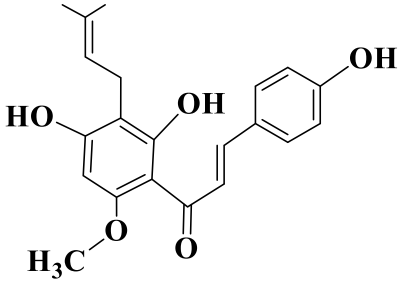	Xanthohumol
7	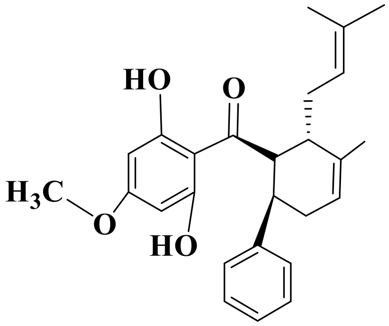	Panduretin A

8	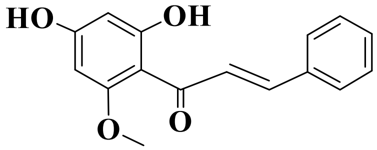	Cardamonin
9	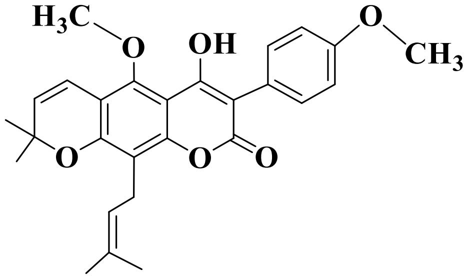	Lonchocarpin
10	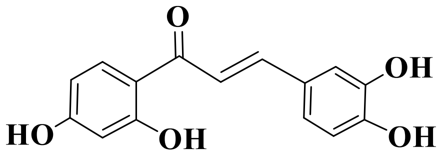	Butein
11	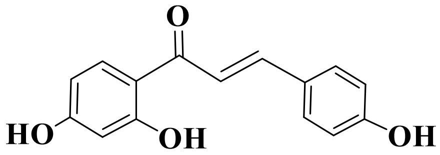	Isoliquiritigenin
12	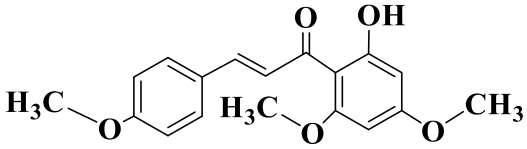	Flavokawain
13	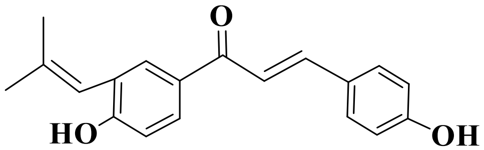	Isobavachalcone
Xanthohumol acyl derivatives		
14-20	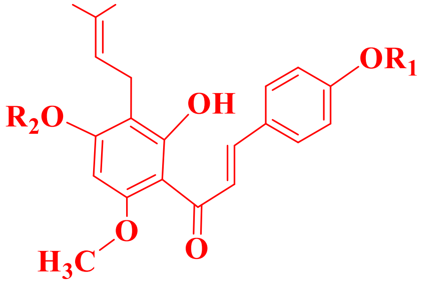	14.R_1=$COCH_3$, R_2=H;<br>15.R_1,R_2=$COCH_3$;<br>16.R_1=$CO(CH_2)_8CH_3$, R_2=H;<br>17. R_1=$CO(CH_2)_{10}CH_3$, R_2=H;<br>18.R_1,R_2= $CO(CH_2)_{10}CH_3$;<br>19.R_1=$COC(CH_3)_3$;<br>20.R_1, R_2=$COC(CH_3)_3$.
Xanthohumol derivatives with cyclized prenyl group (21-26)		
21	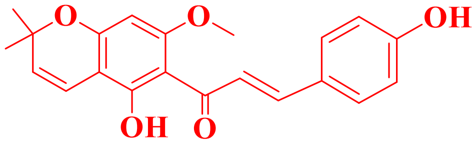	

22	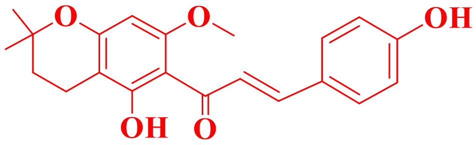		
23	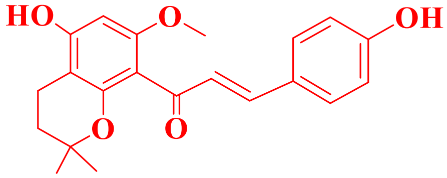		
24	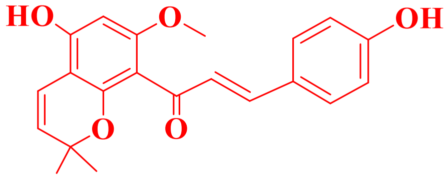		
25	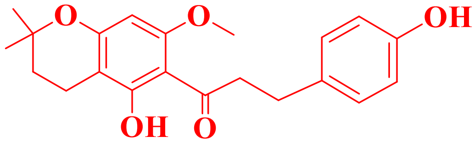		
26	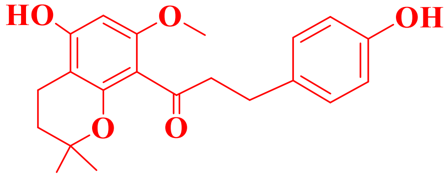		
Chalcone derivatives containing diaryl ether moiety			
27-42	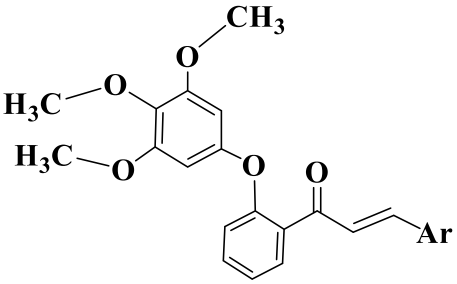	**Ar**<br>27. 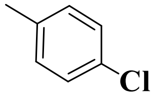	28. 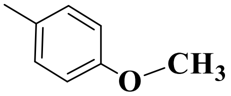
		29. 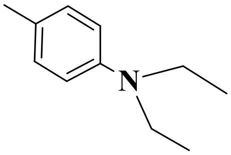	30. 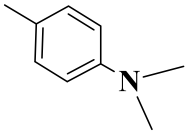
		31. 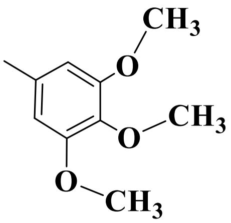	32. 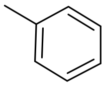
		33. 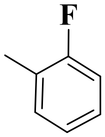	34. 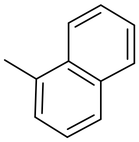

		35. 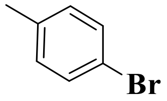	36. 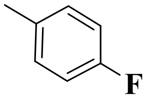
		37. 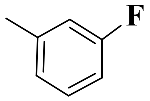	38. 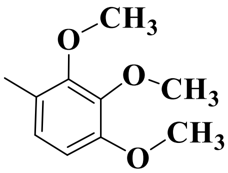
		39. 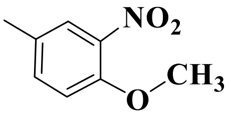	40. 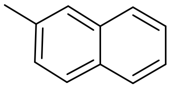
		41. 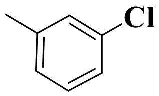	42. 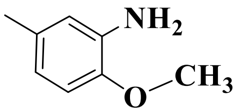
Chalcone derivatives containing sulfonamide moiety			
43-54	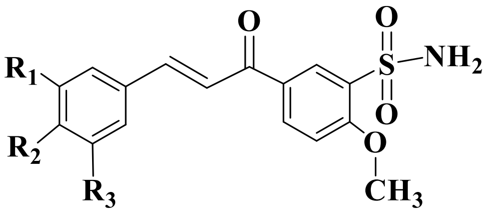	43-48 R_2-43. H; 44.Cl; 45.CH_3; 46.OCH_3, 47.R_1,R_2,R_3=OCH_3; 48. R_2,R_3=CH_2O_2	
	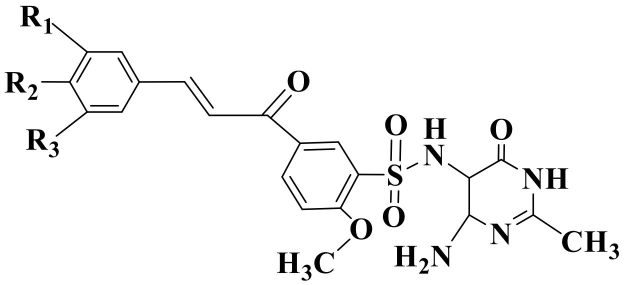	49-54 R_2-49. H; 50.Cl; 51.CH_3; 52.OCH_3; 53.R_1,R_2,R_3=OCH_3; 54. R_2,R_3=CH_2O_2	
55-62	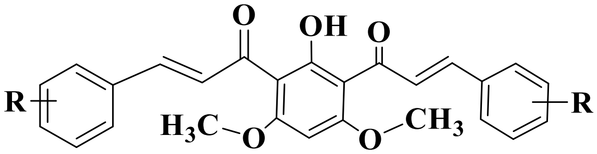	55.R=H; 56.R=2-F; 57.R=3-F; 58.R=4-F; 59.R=2,3-diF; 60.R=2,4-diF; 61.R=2,5-diF; 62.R=3,4-diF	
Aminochalcones			
63-80	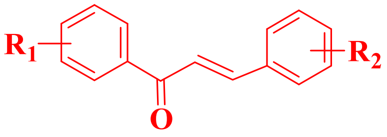	63.R_1=2-NH_2, R_2=H;<br>64.R_1=2-NH_2, R_2=4-CH_2-CH_3;<br>65.R_1=2-NH_2, R_2=4-COOH;<br>66.R_1=2-NH_2, R_2=4-NO_2;<br>67.R_1=2-NH_2, R_2=4-$OCH_2C_6H_5$;<br>68.R_1=2-NH_2, R_2=3-OMe-4$OCH_2C_6H_5$;<br>69.R_1=3-NH_2, R_2=H;<br>70.R_1=3-NH_2, R_2=4-CH_2-CH_3;<br>71.R_1=3-NH_2, R_2=4-COOH;	

		72.R_1=3-NH_2, R_2=4-NO_2;<br>73.R_1=3-NH_2, R_2=4-$OCH_2C_6H_5$;<br>74.R_1=3-NH_2, R_2=3-OMe-4$OCH_2C_6H_5$;<br>75.R_1=4-NH_2, R_2=H;<br>76.R_1=4-NH_2, R_2=4-CH_2-CH_3;<br>77.R_1=4-NH_2, R_2=4-COOH;<br>78.R_1=4-NH_2, R_2=4-NO_2;<br>79.R_1=4-NH_2, R_2=4-$OCH_2C_6H_5$;<br>80.R_1=4-NH_2, R_2=3-OMe-4$OCH_2C_6H_5$;
81-<br>91	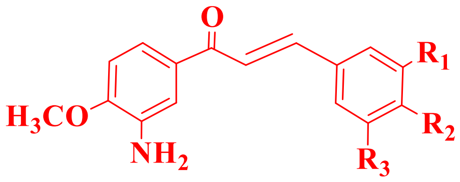	81.R_1=R_2=R_3=H;<br>82.R_1=R_3=H, R_2=OCH_3;<br>83.R_1=R_2=H, R_3=OCH_3;<br>84.R_1=R_3=OCH_3, R_2=H;<br>85.R_1=H, R_2=R_3=OCH_3;<br>86.R_1=R_2=R_3=OCH_3;
	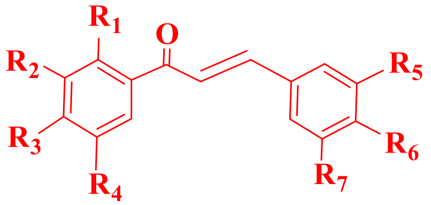	87.R_1=R_7=H; R_2=R_3=R_4=R_6=OCH_3,<br>R_5=NH_2;<br>88.R_1=R_5=R_7=H, R_2=R_3=R_4=OCH_3,<br>R_6=NH_2;<br>89.R_1=R_4=OCH_3, R_2=R_3=R_5=R_7=H,<br>R_6=NH_2;<br>90.R_1=R_4=R_6=OCH_3, R_2=R_3=R_7=H,<br>R_5=NH_2;
	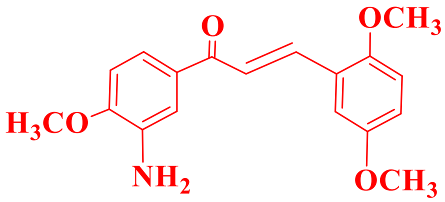	91
92-<br>103	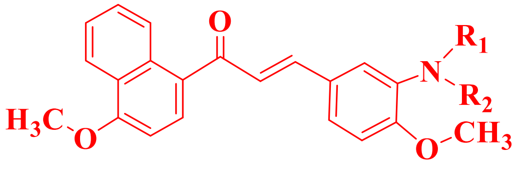	92.R_1=R_2=H;<br>93.R_1=n-propyl, R_2=H;<br>94.R_1=n-butyl, R_2=H;<br>95.R_1= 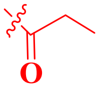 , R_2=H;<br>96.R_1= 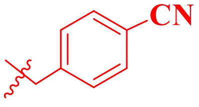 , R_2=H;<br>97.R_1= 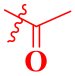 , R_2=H;<br>98.R_1=CH_3, R_2=H;<br>99.R_1=R_2= 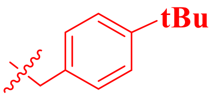 ;

		100.R_1= 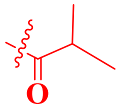 , R_2=H;<br>101.R_1= 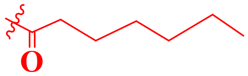 , R_2=H;<br>102.R_1= 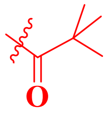 , R_2=H;<br>103.R_1= 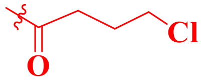 , R_2=H;
Imidazole –chalcone derivatives		
104-121	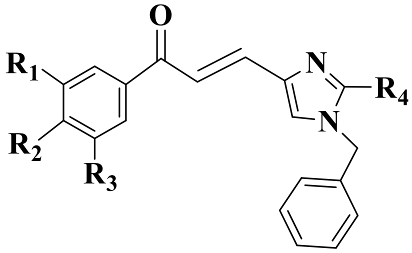	104.R_2=Br, R_1=R_3=H, R_4=SMe;<br>105.R_2=F, R_1=R_3=H, R_4=SMe;<br>106.R_2=Cl, R_1=R_3=H, R_4=SMe;<br>107.R_2=NO_2, R_1=R_3=H, R_4=SMe;<br>108. R_2=OPh, R_1=R_3=H, R_4=SMe;<br>109. R_2=CH_3, R_1=R_3=H, R_4=SMe;<br>110.R_1,R_2=Ph, R_3=H, R_4=SMe;<br>111.R_2=OCH_3, R_1=R_3=H, R_4=SMe;<br>112.R_1=R_2=R_3=OCH_3, R_4=SMe;<br>113.R_2=Br, R_1=R_3=H, R_4=SEt;<br>114.R_2=F, R_1=R_3=H, R_4=SEt;<br>115.R_2=Cl, R_1=R_3=H, R_4=SEt;<br>116.R_2=NO_2, R_1=R_3=H, R_4=SEt;<br>117. R_2=OPh, R_1=R_3=H, R_4=SEt;<br>118. R_2=CH_3, R_1=R_3=H, R_4=SEt;<br>119.R_1,R_2=Ph, R_3=H, R_4=SEt;<br>120.R_2=OCH_3, R_1=R_3=H, R_4=SEt;<br>121.R_1=R_2=R_3=OCH_3, R_4=SEt;
Pyrazole-chalcone derivatives		
122 - 130	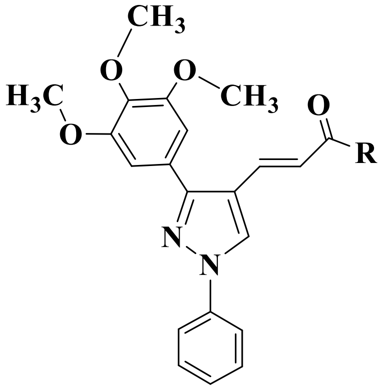	122. R= 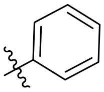 123.R= 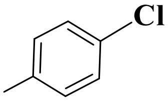<br>124.R= 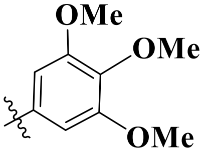 125.R= 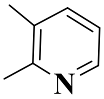<br>126.R= 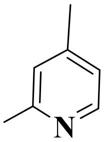 127.R= 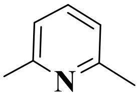<br>128.R= 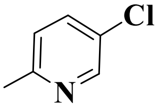 129.R= 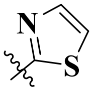

		130.R= 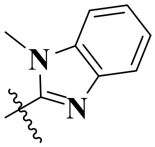
131-172	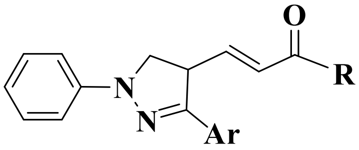	131.Ar= 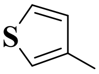, R= 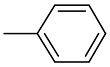;<br>132.Ar= 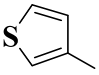, R= 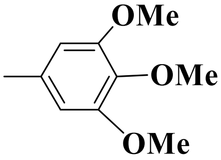;<br>133.Ar= 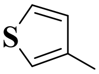, R= 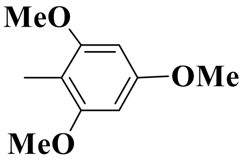;<br>134.Ar= 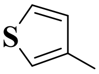, R= 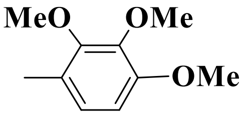;<br>135.Ar= 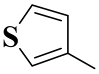, R= 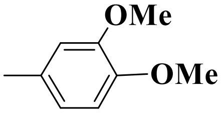;<br>136.Ar= 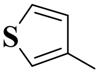, R= 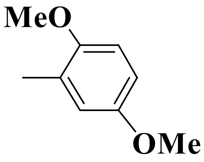;<br>137.Ar= 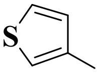, R= 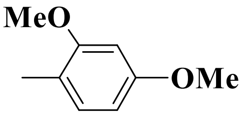;<br>138.Ar= 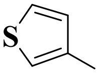, R= 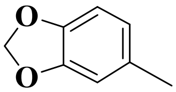<br>139.Ar= 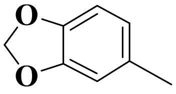, R= 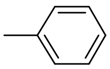;<br>140.Ar= 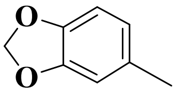, R= 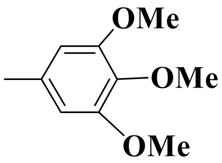;

		141.Ar= 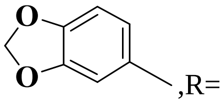 ,R= 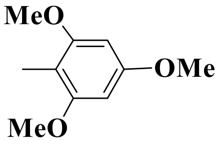 ;<br>142.Ar= 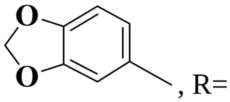 , R= 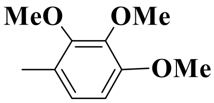 ;<br>143.Ar= 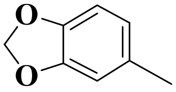 , R= 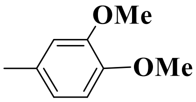<br>144.Ar= 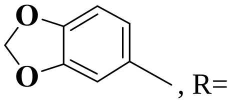 , R= 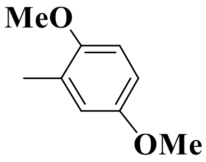<br>145.Ar= 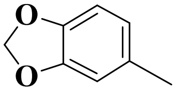 , R= 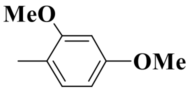 ;<br>146.Ar= 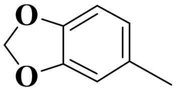 , R= 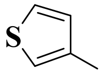 ;<br>147.Ar= 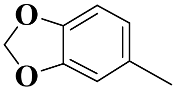 , R= 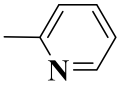 ;<br>148.Ar= 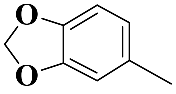 , R= 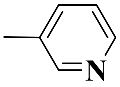 ;<br>149.Ar= 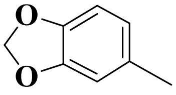 , R= 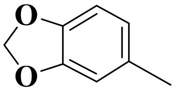 ;<br>150.Ar= 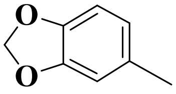 , R= 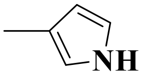 ;<br>151.Ar= 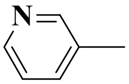 , R= 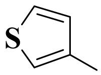 ;<br>152.Ar= 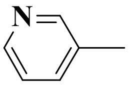 , R= 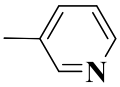 ;

		153.Ar= 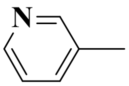 , R= 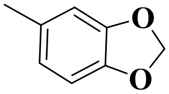 ;<br>154.Ar= 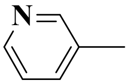 , R= 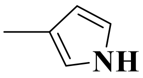 ;<br>155.Ar= 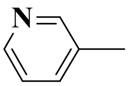 , R= 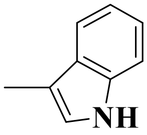 ;<br>156.Ar= 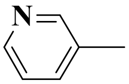 ,R= 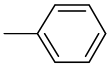 ;<br>157.Ar= 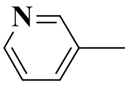 , R= 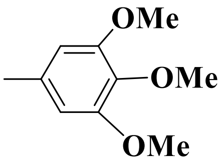 ;<br>158.Ar= 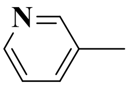 , R= 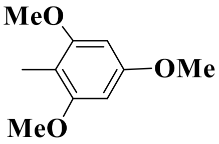 ;<br>159.Ar= 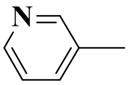 , R= 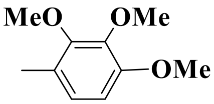 ;<br>160.Ar= 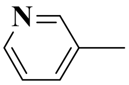 , R= 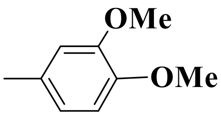 ;<br>161.Ar= 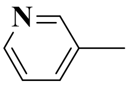 , R= 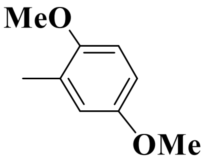 ;<br>162.Ar= 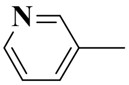 , R= 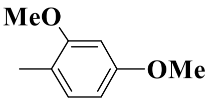 ;<br>163.Ar= 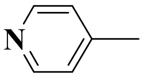 , R= 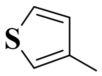 ;<br>164.Ar= 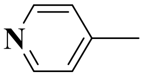 ,R= 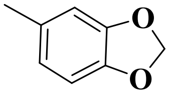 ;<br>165.Ar= 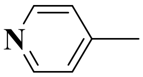 , R= 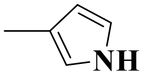 ;<br>166.Ar= 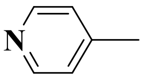 , R= 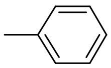 ;

		167.Ar= 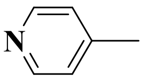 ,R= 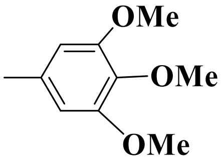 ;<br>168.Ar= 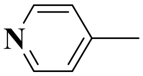 , R= 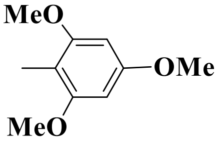 ;<br>169.Ar= 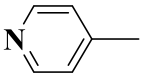 , R= 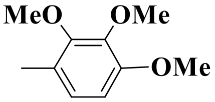 ;<br>170.Ar= 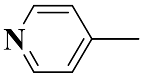 , R= 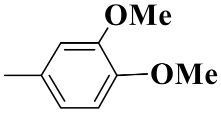 ;<br>171.Ar= 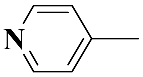 , R= 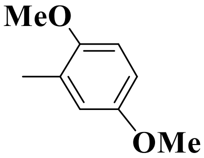 ;<br>172.Ar= 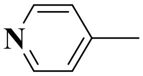 , R= 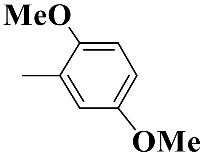
Tetrazole-chalcone derivatives		
173-180	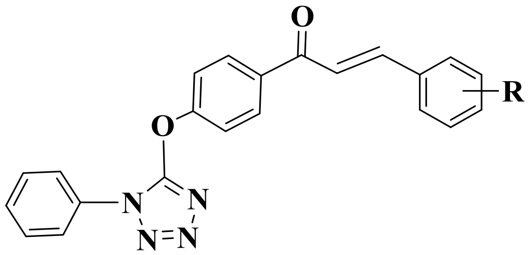	173.R=H; 174.R=3,4-$(OCH_3)_2$; 175.R=3,4,5-$(OCH_3)_3$; 176.R=4-Cl; 177.R=2-Br; 178.4-Br; 179.R=4-F; 180.R=2-NO_2
Thiazole-chalcone derivatives		
181-186	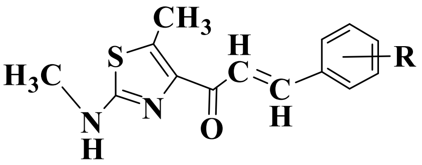	181.R=4-OCH_3; 182.R=4-CH_3; 183.R=H; 184.R=4-F; 185.R=4-Cl; 186.R=2,4-diCl

187-196	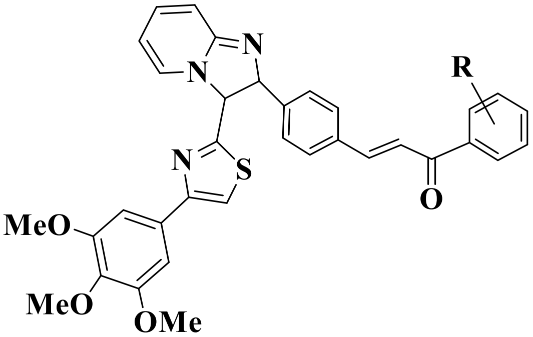	187.R=H; 188.R=3,4,5-$(OCH_3)_3$; 189.R=3,5-$(OCH_3)_2$; 190.R=4-OCH_3; 191.R=4-Cl; 192.R=4-Br; 193.R=4-NO_2; 194.R=3,5-$(NO_2)_2$; 195.R=4-CN; 196.R=4-CH_3
Tetrazole-chalcone derivatives		
197	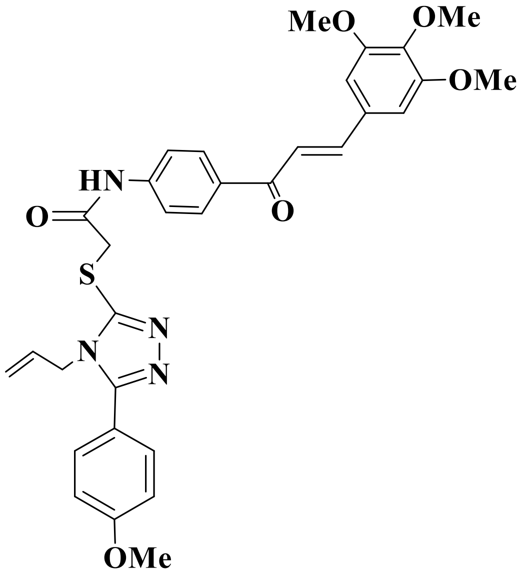	1,2,4-triazole chlacone derivative
198-206	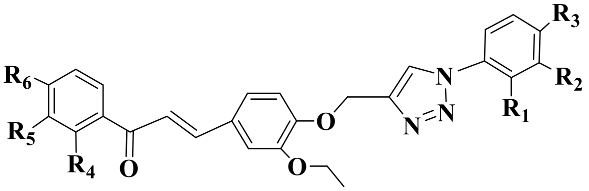	198.R_1=OH, R_2=R_3=R_4=H, R_5=R_6=OMe; 199. R_1=OH, R_2=R_3=R_4=R_5=H, R_6=Br; 200. R_1=OH, R_2=R_3=R_4=R_6=H, R_5=OH; 201. R_1=OH, R_2=R_4= H, R_3=Me,R_5, R_6=OMe; 202. R_1=OH, R_2=R_4=R_5=H, R_3=Me, R_6=Br; 203. R_1=OH, R_2=R_4=R_6=H, R_3=Me, R_5=OH; 204. R_1=OH, R_2=R_4 =H, R_3=Cl, R_5=R_6=OMe; 205. R_1=OH, R_2=R_4=R_5=H, R_3=Cl, R_6=Br; 206. R_1=OH, R_2=R_4=R_6=H, R_3=Cl, R_5=OH.
