# Supplementary material for: Anticancer Activity of Natural and Synthetic Chalcones
Source: Int J Mol Sci. 2021 Oct 20;22(21):11306. doi: 10.3390/ijms222111306 (PMC8582663; doi:10.3390/ijms222111306)
Supplement: Supplementary file 1 [file ijms-22-11306-s001.zip › Table S2 compounds names.pdf]

Table S2. Compounds names.

Comp.	Name	Chemical Formula
1	(E)-3-(4-hydroxy-2-methoxy-5-vinylphenyl)-1-(4-hydroxyphenyl)prop-2-en-1-one	$C_{18}H_{16}O_4$
2	(E)-chalcone	$C_{15}H_{12}O$
3	(E)-3-(3,4-dihydroxy-2-methoxyphenyl)-1-(4-hydroxyphenyl)prop-2-en-1-one	$C_{16}H_{14}O_5$
4	(E)-3-(4-hydroxy-2-methoxy-3-(3-methylbut-2-en-1-yl)phenyl)-1-(4-hydroxyphenyl)prop-2-en-1-one	$C_{21}H_{22}O_4$
5	(E)-3-(3,4-dihydroxy-2-methoxyphenyl)-1-(4-hydroxy-3-(3-methylbut-2-en-1-yl)phenyl)prop-2-en-1-one	$C_{21}H_{22}O_5$
6	(E)-1-(2,4-dihydroxy-6-methoxy-3-(3-methylbut-2-en-1-yl)phenyl)-3-(4-hydroxyphenyl)prop-2-en-1-one	$C_{21}H_{22}O_5$
7	(2,6-dihydroxy-4-methoxyphenyl)((1R,2R,3S)-4-methyl-3-(3-methylbut-2-en-1-yl)-1,2,3,6-tetrahydro-[1,1'-biphenyl]-2-yl)methanone	$C_{26}H_{30}O_4$
8	(E)-1-(2,4-dihydroxy-6-methoxyphenyl)-3-phenylprop-2-en-1-one	$C_{16}H_{14}O_4$
9	4-hydroxy-5-methoxy-3-(4-methoxyphenyl)-8,8-dimethyl-10-(3-methylbut-2-en-1-yl)-2H,8H-pyrano[3,2-g]chromen-2-one	$C_{27}H_{28}O_6$
10	(E)-1-(2,4-dihydroxyphenyl)-3-(3,4-dihydroxyphenyl)prop-2-en-1-one	$C_{15}H_{12}O_5$
11	(E)-1-(2,4-dihydroxyphenyl)-3-(4-hydroxyphenyl)prop-2-en-1-one	$C_{15}H_{12}O_4$
12	(E)-1-(2-hydroxy-4,6-dimethoxyphenyl)-3-(4-methoxyphenyl)prop-2-en-1-one	$C_{18}H_{18}O_5$
13	(E)-1-(4-hydroxy-3-(2-methylprop-1-en-1-yl)phenyl)-3-(4-hydroxyphenyl)prop-2-en-1-one	$C_{19}H_{18}O_3$
14	(E)-3-(4-acetylphenyl)-1-(2,4-dihydroxy-6-methoxy-3-(3-methylbut-2-en-1-yl)phenyl)prop-2-en-1-one	$C_{23}H_{24}O_5$
15	(E)-1-(4-acetyl-2-hydroxy-6-methoxy-3-(3-methylbut-2-en-1-yl)phenyl)-3-(4-acetylphenyl)prop-2-en-1-one	$C_{25}H_{26}O_5$
16	(E)-1-(4-(3-(2,4-dihydroxy-6-methoxy-3-(3-methylbut-2-en-1-yl)phenyl)-3-oxoprop-1-en-1-yl)phenyl)decan-1-one	$C_{31}H_{40}O_5$
17	(E)-1-(4-(3-(2,4-dihydroxy-6-methoxy-3-(3-methylbut-2-en-1-yl)phenyl)-3-oxoprop-1-en-1-yl)phenyl)dodecan-1-one	$C_{33}H_{44}O_5$
18	1-(4-((E)-3-(4-dodecanoylphenyl)acryloyl)-3-hydroxy-5-methoxy-2-(3-methylbut-2-en-1-yl)phenyl)dodeca-2,4,6,8,10-pentaen-1-one	$C_{45}H_{56}O_5$
19	(E)-1-(2,4-dihydroxy-6-methoxy-3-(3-methylbut-2-en-1-yl)phenyl)-3-(4-pivaloylphenyl)prop-2-en-1-one	$C_{26}H_{30}O_5$
20	(E)-1-(3-hydroxy-5-methoxy-2-(3-methylbut-2-en-1-yl)-4-(3-(4-pivaloylphenyl)acryloyl)phenyl)-2-methyl-1-oxopropan-2-ide	$C_{30}H_{35}O_5$
21	(E)-1-(5-hydroxy-7-methoxy-2,2-dimethyl-2H-chromen-6-yl)-3-(4-hydroxyphenyl)prop-2-en-1-one	$C_{21}H_{20}O_5$
22	(E)-1-(5-hydroxy-7-methoxy-2,2-dimethylchroman-6-yl)-3-(4-hydroxyphenyl)prop-2-en-1-one	$C_{21}H_{22}O_5$

23	(E)-1-(5-hydroxy-7-methoxy-2,2-dimethylchroman-8-yl)-3-(4-hydroxyphenyl)prop-2-en-1-one	$C_{21}H_{22}O_5$
24	(E)-1-(5-hydroxy-7-methoxy-2,2-dimethyl-2H-chromen-8-yl)-3-(4-hydroxyphenyl)prop-2-en-1-one	$C_{21}H_{20}O_5$
25	1-(5-hydroxy-7-methoxy-2,2-dimethylchroman-6-yl)-3-(4-hydroxyphenyl)propan-1-one	$C_{21}H_{24}O_5$
26	1-(5-hydroxy-7-methoxy-2,2-dimethylchroman-8-yl)-3-(4-hydroxyphenyl)propan-1-one	$C_{21}H_{24}O_5$
27	(E)-3-(4-chlorophenyl)-1-(2-(3,4,5-trimethoxyphenoxy)phenyl)prop-2-en-1-one	$C_{24}H_{21}ClO_5$
28	(E)-3-(4-methoxyphenyl)-1-(2-(3,4,5-trimethoxyphenoxy)phenyl)prop-2-en-1-one	$C_{25}H_{24}O_6$
29	(E)-3-(4-(diethylamino)phenyl)-1-(2-(3,4,5-trimethoxyphenoxy)phenyl)prop-2-en-1-one	$C_{28}H_{31}NO_5$
30	(E)-3-(4-(dimethylamino)phenyl)-1-(2-(3,4,5-trimethoxyphenoxy)phenyl)prop-2-en-1-one	$C_{26}H_{27}NO_5$
31	(E)-1-(2-(3,4,5-trimethoxyphenoxy)phenyl)-3-(3,4,5-trimethoxyphenyl)prop-2-en-1-one	$C_{27}H_{28}O_8$
32	(E)-3-phenyl-1-(2-(3,4,5-trimethoxyphenoxy)phenyl)prop-2-en-1-one	$C_{24}H_{22}O_5$
33	(E)-3-(2-fluorophenyl)-1-(2-(3,4,5-trimethoxyphenoxy)phenyl)prop-2-en-1-one	$C_{24}H_{21}FO_5$
34	(E)-3-(naphthalen-1-yl)-1-(2-(3,4,5-trimethoxyphenoxy)phenyl)prop-2-en-1-one	$C_{28}H_{24}O_5$
35	(E)-3-(4-bromophenyl)-1-(2-(3,4,5-trimethoxyphenoxy)phenyl)prop-2-en-1-one	$C_{24}H_{21}BrO_5$
36	(E)-3-(4-fluorophenyl)-1-(2-(3,4,5-trimethoxyphenoxy)phenyl)prop-2-en-1-one	$C_{24}H_{21}FO_5$
37	(E)-3-(3-fluorophenyl)-1-(2-(3,4,5-trimethoxyphenoxy)phenyl)prop-2-en-1-one	$C_{24}H_{21}FO_5$
38	(E)-1-(2-(3,4,5-trimethoxyphenoxy)phenyl)-3-(2,3,4-trimethoxyphenyl)prop-2-en-1-one	$C_{27}H_{28}O_8$
39	(E)-3-(4-methoxy-3-nitrophenyl)-1-(2-(3,4,5-trimethoxyphenoxy)phenyl)prop-2-en-1-one	$C_{25}H_{23}NO_8$
40	(E)-3-(naphthalen-2-yl)-1-(2-(3,4,5-trimethoxyphenoxy)phenyl)prop-2-en-1-one	$C_{28}H_{24}O_5$
41	(E)-3-(3-chlorophenyl)-1-(2-(3,4,5-trimethoxyphenoxy)phenyl)prop-2-en-1-one	$C_{24}H_{21}ClO_5$
42	(E)-3-(3-amino-4-methoxyphenyl)-1-(2-(3,4,5-trimethoxyphenoxy)phenyl)prop-2-en-1-one	$C_{25}H_{25}NO_6$
43	5-cinnamoyl-2-methoxybenzenesulfonamide	$C_{16}H_{15}NO_4S$
44	(E)-5-(3-(4-chlorophenyl)acryloyl)-2-methoxybenzenesulfonamide	$C_{16}H_{14}ClNO_4S$
45	(E)-2-methoxy-5-(3-(p-tolyl)acryloyl)benzenesulfonamide	$C_{17}H_{17}NO_4S$
46	(E)-2-methoxy-5-(3-(4-methoxyphenyl)acryloyl)benzenesulfonamide	$C_{17}H_{17}NO_5S$

47	(E)-2-methoxy-5-(3-(3,4,5-trimethoxyphenyl)acryloyl)benzenesulfonamide	$C_{19}H_{21}NO_7S$
48	(E)-5-(3-(benzo[d][1,3]dioxol-5-yl)acryloyl)-2-methoxybenzenesulfonamide	$C_{17}H_{15}NO_6S$
49	N-(4-amino-2-methyl-6-oxo-1,4,5,6-tetrahydropyrimidin-5-yl)-5-cinnamoyl-2-methoxybenzenesulfonamide	$C_{21}H_{22}N_4O_5S$
50	(E)-N-(4-amino-2-methyl-6-oxo-1,4,5,6-tetrahydropyrimidin-5-yl)-5-(3-(4-chlorophenyl)acryloyl)-2-methoxybenzenesulfonamide	$C_{21}H_{21}ClN_4O_5S$
51	(E)-N-(4-amino-2-methyl-6-oxo-1,4,5,6-tetrahydropyrimidin-5-yl)-2-methoxy-5-(3-(p-tolyl)acryloyl)benzenesulfonamide	$C_{22}H_{24}N_4O_5S$
52	(E)-N-(4-amino-2-methyl-6-oxo-1,4,5,6-tetrahydropyrimidin-5-yl)-2-methoxy-5-(3-(p-tolyl)acryloyl)benzenesulfonamide	$C_{22}H_{24}N_4O_6S$
53	(E)-N-(4-amino-2-methyl-6-oxo-1,4,5,6-tetrahydropyrimidin-5-yl)-2-methoxy-5-(3-(p-tolyl)acryloyl)benzenesulfonamide	$C_{24}H_{28}N_4O_8S$
54	(E)-N-(4-amino-2-methyl-6-oxo-1,4,5,6-tetrahydropyrimidin-5-yl)-2-methoxy-5-(3-(p-tolyl)acryloyl)benzenesulfonamide	$C_{22}H_{22}N_4O_7S$
55	(2E,2'E)-1,1'-(2-hydroxy-4,6-dimethoxy-1,3-phenylene)bis(3-phenylprop-2-en-1-one)	$C_{26}H_{22}O_5$
56	(2E,2'E)-1,1'-(2-hydroxy-4,6-dimethoxy-1,3-phenylene)bis(3-(2-fluorophenyl)prop-2-en-1-one)	$C_{26}H_{20}F_2O_5$
57	(2E,2'E)-1,1'-(2-hydroxy-4,6-dimethoxy-1,3-phenylene)bis(3-(3-fluorophenyl)prop-2-en-1-one)	$C_{26}H_{20}F_2O_5$
58	(2E,2'E)-1,1'-(2-hydroxy-4,6-dimethoxy-1,3-phenylene)bis(3-(4-fluorophenyl)prop-2-en-1-one)	$C_{26}H_{20}F_2O_5$
59	(2E,2'E)-1,1'-(2-hydroxy-4,6-dimethoxy-1,3-phenylene)bis(3-(2,3-difluorophenyl)prop-2-en-1-one)	$C_{26}H_{18}F_4O_5$
60	(2E,2'E)-1,1'-(2-hydroxy-4,6-dimethoxy-1,3-phenylene)bis(3-(2,4-difluorophenyl)prop-2-en-1-one)	$C_{26}H_{18}F_4O_5$
61	(2E,2'E)-1,1'-(2-hydroxy-4,6-dimethoxy-1,3-phenylene)bis(3-(2,5-difluorophenyl)prop-2-en-1-one)	$C_{26}H_{18}F_4O_5$
62	(2E,2'E)-1,1'-(2-hydroxy-4,6-dimethoxy-1,3-phenylene)bis(3-(3,4-difluorophenyl)prop-2-en-1-one)	$C_{26}H_{18}F_4O_5$
63	(E)-1-(2-aminophenyl)-3-phenylprop-2-en-1-one	$C_{15}H_{13}NO$
64	(E)-1-(2-aminophenyl)-3-(4-ethylphenyl)prop-2-en-1-one	$C_{17}H_{17}NO$
65	(E)-4-(3-(2-aminophenyl)-3-oxoprop-1-en-1-yl)benzoic acid	$C_{16}H_{13}NO_3$
66	(E)-1-(2-aminophenyl)-3-(2-nitrophenyl)prop-2-en-1-one	$C_{15}H_{12}N_2O_3$
67	(E)-1-(2-aminophenyl)-3-(2-(benzyloxy)phenyl)prop-2-en-1-one	$C_{22}H_{19}NO_2$
68	(E)-1-(2-aminophenyl)-3-(4-(benzyloxy)-3-methoxyphenyl)prop-2-en-1-one	$C_{23}H_{21}NO_3$
69	(E)-1-(3-aminophenyl)-3-phenylprop-2-en-1-one	$C_{15}H_{13}NO$
70	(E)-1-(3-aminophenyl)-3-(4-ethylphenyl)prop-2-en-1-one	$C_{17}H_{17}NO$
71	(E)-4-(3-(3-aminophenyl)-3-oxoprop-1-en-1-yl)benzoic acid	$C_{16}H_{13}NO_3$
72	(E)-1-(3-aminophenyl)-3-(4-nitrophenyl)prop-2-en-1-one	$C_{15}H_{12}N_2O_3$
73	(E)-1-(3-aminophenyl)-3-(4-(benzyloxy)phenyl)prop-2-en-1-one	$C_{22}H_{19}NO_2$

74	(E)-1-(3-aminophenyl)-3-(4-(benzyloxy)-3-methoxyphenyl)prop-2-en-1-one	$C_{23}H_{21}NO_3$
75	(E)-1-(4-aminophenyl)-3-phenylprop-2-en-1-one	$C_{15}H_{13}NO$
76	(E)-1-(4-aminophenyl)-3-(4-ethylphenyl)prop-2-en-1-one	$C_{17}H_{17}NO$
77	(E)-4-(3-(4-aminophenyl)-3-oxoprop-1-en-1-yl)benzoic acid	$C_{16}H_{13}NO_3$
78	(E)-1-(4-aminophenyl)-3-(4-nitrophenyl)prop-2-en-1-one	$C_{15}H_{12}N_2O_3$
79	(E)-1-(4-aminophenyl)-3-(4-(benzyloxy)phenyl)prop-2-en-1-one	$C_{22}H_{19}NO_2$
80	(E)-1-(4-aminophenyl)-3-(4-(benzyloxy)-3-methoxyphenyl)prop-2-en-1-one	$C_{23}H_{21}NO_3$
81	(E)-1-(3-amino-4-methoxyphenyl)-3-phenylprop-2-en-1-one	$C_{16}H_{15}NO_2$
82	(E)-1-(3-amino-4-methoxyphenyl)-3-(4-methoxyphenyl)prop-2-en-1-one	$C_{17}H_{17}NO_3$
83	(E)-1-(3-amino-4-methoxyphenyl)-3-(3-methoxyphenyl)prop-2-en-1-one	$C_{17}H_{17}NO_3$
84	(E)-1-(3-amino-4-methoxyphenyl)-3-(3,5-dimethoxyphenyl)prop-2-en-1-one	$C_{18}H_{19}NO_4$
85	(E)-1-(3-amino-4-methoxyphenyl)-3-(3,5-dimethoxyphenyl)prop-2-en-1-one	$C_{18}H_{19}NO_4$
86	(E)-1-(3-amino-4-methoxyphenyl)-3-(3,4,5-trimethoxyphenyl)prop-2-en-1-one	$C_{19}H_{21}NO_5$
87	(E)-3-(3-amino-4-methoxyphenyl)-1-(3,4,5-trimethoxyphenyl)prop-2-en-1-one	$C_{19}H_{21}NO_5$
88	(E)-3-(4-aminophenyl)-1-(3,4,5-trimethoxyphenyl)prop-2-en-1-one	$C_{18}H_{19}NO_4$
89	(E)-3-(4-aminophenyl)-1-(2,5-dimethoxyphenyl)prop-2-en-1-one	$C_{17}H_{17}NO_3$
90	(E)-3-(3-amino-4-methoxyphenyl)-1-(2,5-dimethoxyphenyl)prop-2-en-1-one	$C_{18}H_{19}NO_4$
91	(E)-1-(3-amino-4-methoxyphenyl)-3-(2,5-dimethoxyphenyl)prop-2-en-1-one	$C_{18}H_{19}NO_4$
92	(E)-3-(3-amino-4-methoxyphenyl)-1-(4-methoxynaphthalen-1-yl)prop-2-en-1-one	$C_{21}H_{19}NO_3$
93	(E)-3-(4-methoxy-3-(propylamino)phenyl)-1-(4-methoxynaphthalen-1-yl)prop-2-en-1-one	$C_{24}H_{25}NO_3$
94	(E)-3-(3-(butylamino)-4-methoxyphenyl)-1-(4-methoxynaphthalen-1-yl)prop-2-en-1-one	$C_{25}H_{27}NO_3$
95	(E)-N-(2-methoxy-5-(3-(4-methoxynaphthalen-1-yl)-3-oxoprop-1-en-1-yl)phenyl)propionamide	$C_{24}H_{23}NO_4$
96	(E)-4-(((2-methoxy-5-(3-(4-methoxynaphthalen-1-yl)-3-oxoprop-1-en-1-yl)phenyl)amino)methyl)benzonitrile	$C_{29}H_{24}N_2O_3$
97	(E)-N-(2-methoxy-5-(3-(4-methoxynaphthalen-1-yl)-3-oxoprop-1-en-1-yl)phenyl)acetamide	$C_{23}H_{21}NO_4$
98	(E)-3-(4-methoxy-3-(methylamino)phenyl)-1-(4-methoxynaphthalen-1-yl)prop-2-en-1-one	$C_{22}H_{21}NO_3$
99	(E)-3-(3-((4-(tert-butyl)benzyl)amino)-4-methoxyphenyl)-1-(4-methoxynaphthalen-1-yl)prop-2-en-1-one	$C_{32}H_{33}NO_3$

100	(E)-N-(2-methoxy-5-(3-(4-methoxynaphthalen-1-yl)-3-oxoprop-1-en-1-yl)phenyl)isobutyramide	$C_{25}H_{25}NO_4$
101	(E)-N-(2-methoxy-5-(3-(4-methoxynaphthalen-1-yl)-3-oxoprop-1-en-1-yl)phenyl)heptanamide	$C_{28}H_{31}NO_4$
102	(E)-N-(2-methoxy-5-(3-(4-methoxynaphthalen-1-yl)-3-oxoprop-1-en-1-yl)phenyl)pivalamide	$C_{26}H_{27}NO_4$
103	(E)-4-chloro-N-(2-hydroxy-5-(3-(4-methoxynaphthalen-1-yl)-3-oxoprop-1-en-1-yl)phenyl)butanamide	$C_{24}H_{22}ClNO_4$
104	(E)-3-(1-benzyl-2-(methylthio)-1H-imidazol-4-yl)-1-(4-bromophenyl)prop-2-en-1-one	$C_{20}H_{17}BrN_2OS$
105	(E)-3-(1-benzyl-2-(methylthio)-1H-imidazol-4-yl)-1-(4-fluorophenyl)prop-2-en-1-one	$C_{20}H_{17}FN_2OS$
106	(E)-3-(1-benzyl-2-(methylthio)-1H-imidazol-4-yl)-1-(4-chlorophenyl)prop-2-en-1-one	$C_{20}H_{17}ClN_2OS$
107	(E)-3-(1-benzyl-2-(methylthio)-1H-imidazol-4-yl)-1-(4-nitrophenyl)prop-2-en-1-one	$C_{20}H_{17}N_3O_3S$
108	(E)-3-(1-benzyl-2-(methylthio)-1H-imidazol-4-yl)-1-(4-phenoxyphenyl)prop-2-en-1-one	$C_{26}H_{22}N_2O_2S$
109	(E)-3-(1-benzyl-2-(methylthio)-1H-imidazol-4-yl)-1-(p-tolyl)prop-2-en-1-one	$C_{21}H_{20}N_2OS$
110	(E)-1-([1,1':2',1''-terphenyl]-4'-yl)-3-(1-benzyl-2-(methylthio)-1H-imidazol-4-yl)prop-2-en-1-one	$C_{32}H_{26}N_2OS$
111	(E)-3-(1-benzyl-2-(methylthio)-1H-imidazol-4-yl)-1-(4-methoxyphenyl)prop-2-en-1-one	$C_{21}H_{20}N_2O_2S$
112	(E)-3-(1-benzyl-2-(methylthio)-1H-imidazol-4-yl)-1-(3,4,5-trimethoxyphenyl)prop-2-en-1-one	$C_{23}H_{24}N_2O_4S$
113	(E)-3-(1-benzyl-2-(ethylthio)-1H-imidazol-4-yl)-1-(4-bromophenyl)prop-2-en-1-one	$C_{21}H_{19}BrN_2OS$
114	(E)-3-(1-benzyl-2-(ethylthio)-1H-imidazol-4-yl)-1-(4-fluorophenyl)prop-2-en-1-one	$C_{21}H_{19}FN_2OS$
115	(E)-3-(1-benzyl-2-(ethylthio)-1H-imidazol-4-yl)-1-(4-chlorophenyl)prop-2-en-1-one	$C_{21}H_{19}ClN_2OS$
116	(E)-3-(1-benzyl-2-(ethylthio)-1H-imidazol-4-yl)-1-(4-nitrophenyl)prop-2-en-1-one	$C_{21}H_{19}N_3O_3S$
117	(E)-3-(1-benzyl-2-(ethylthio)-1H-imidazol-4-yl)-1-(4-phenoxyphenyl)prop-2-en-1-one	$C_{27}H_{24}N_2O_2S$
118	(E)-3-(1-benzyl-2-(ethylthio)-1H-imidazol-4-yl)-1-(p-tolyl)prop-2-en-1-one	$C_{22}H_{22}N_2OS$
119	(E)-1-([1,1':2',1''-terphenyl]-4'-yl)-3-(1-benzyl-2-(ethylthio)-1H-imidazol-4-yl)prop-2-en-1-one	$C_{33}H_{28}N_2OS$
120	(E)-3-(1-benzyl-2-(ethylthio)-1H-imidazol-4-yl)-1-(4-methoxyphenyl)prop-2-en-1-one	$C_{22}H_{22}N_2O_2S$
121	(E)-3-(1-benzyl-2-(ethylthio)-1H-imidazol-4-yl)-1-(3,4,5-trimethoxyphenyl)prop-2-en-1-one	$C_{24}H_{26}N_2O_4S$

122	(E)-1-phenyl-3-(1-phenyl-3-(3,4,5-trimethoxyphenyl)-1H-pyrazol-4-yl)prop-2-en-1-one	$C_{27}H_{24}N_2O_4$
123	(E)-1-(4-chlorophenyl)-3-(1-phenyl-3-(3,4,5-trimethoxyphenyl)-1H-pyrazol-4-yl)prop-2-en-1-one	$C_{27}H_{23}ClN_2O_4$
124	(E)-3-(1-phenyl-3-(3,4,5-trimethoxyphenyl)-1H-pyrazol-4-yl)-1-(3,4,5-trimethoxyphenyl)prop-2-en-1-one	$C_{30}H_{30}N_2O_7$
125	(E)-1-(2-methylpyridin-3-yl)-3-(1-phenyl-3-(3,4,5-trimethoxyphenyl)-1H-pyrazol-4-yl)prop-2-en-1-one	$C_{27}H_{25}N_3O_4$
126	(E)-1-(4-methylpyridin-2-yl)-3-(1-phenyl-3-(3,4,5-trimethoxyphenyl)-1H-pyrazol-4-yl)prop-2-en-1-one	$C_{27}H_{25}N_3O_4$
127	(E)-1-(6-methylpyridin-2-yl)-3-(1-phenyl-3-(3,4,5-trimethoxyphenyl)-1H-pyrazol-4-yl)prop-2-en-1-one	$C_{27}H_{25}N_3O_4$
128	(E)-1-(5-chloropyridin-2-yl)-3-(1-phenyl-3-(3,4,5-trimethoxyphenyl)-1H-pyrazol-4-yl)prop-2-en-1-one	$C_{26}H_{22}ClN_3O_4$
129	(E)-3-(1-phenyl-3-(3,4,5-trimethoxyphenyl)-1H-pyrazol-4-yl)-1-(thiazol-2-yl)prop-2-en-1-one	$C_{24}H_{21}N_3O_4S$
130	(E)-1-(1-methyl-1H-benzo[d]imidazol-2-yl)-3-(1-phenyl-3-(3,4,5-trimethoxyphenyl)-1H-pyrazol-4-yl)prop-2-en-1-one	$C_{29}H_{26}N_4O_4$
131	(E)-1-phenyl-3-(1-phenyl-3-(thiophen-3-yl)-4,5-dihydro-1H-pyrazol-4-yl)prop-2-en-1-one	$C_{22}H_{18}N_2OS$
132	(E)-3-(1-phenyl-3-(thiophen-3-yl)-4,5-dihydro-1H-pyrazol-4-yl)-1-(3,4,5-trimethoxyphenyl)prop-2-en-1-one	$C_{25}H_{24}N_2O_4S$
133	(E)-3-(1-phenyl-3-(thiophen-3-yl)-4,5-dihydro-1H-pyrazol-4-yl)-1-(2,4,6-trimethoxyphenyl)prop-2-en-1-one	$C_{25}H_{24}N_2O_4S$
134	(E)-3-(1-phenyl-3-(thiophen-3-yl)-4,5-dihydro-1H-pyrazol-4-yl)-1-(2,3,4-trimethoxyphenyl)prop-2-en-1-one	$C_{25}H_{24}N_2O_4S$
135	(E)-1-(3,4-dimethoxyphenyl)-3-(1-phenyl-3-(thiophen-3-yl)-4,5-dihydro-1H-pyrazol-4-yl)prop-2-en-1-one	$C_{24}H_{22}N_2O_3S$
136	(E)-1-(2,5-dimethoxyphenyl)-3-(1-phenyl-3-(thiophen-3-yl)-4,5-dihydro-1H-pyrazol-4-yl)prop-2-en-1-one	$C_{24}H_{22}N_2O_3S$
137	(E)-1-(2,4-dimethoxyphenyl)-3-(1-phenyl-3-(thiophen-3-yl)-4,5-dihydro-1H-pyrazol-4-yl)prop-2-en-1-one	$C_{24}H_{22}N_2O_3S$
138	(E)-1-(benzo[d][1,3]dioxol-5-yl)-3-(1-phenyl-3-(thiophen-3-yl)-4,5-dihydro-1H-pyrazol-4-yl)prop-2-en-1-one	$C_{23}H_{18}N_2O_3S$
139	(E)-3-(3-(benzo[d][1,3]dioxol-5-yl)-1-phenyl-4,5-dihydro-1H-pyrazol-4-yl)-1-phenylprop-2-en-1-one	$C_{25}H_{20}N_2O_3$
140	(E)-3-(3-(benzo[d][1,3]dioxol-5-yl)-1-phenyl-4,5-dihydro-1H-pyrazol-4-yl)-1-(3,4,5-trimethoxyphenyl)prop-2-en-1-one	$C_{28}H_{26}N_2O_6$
141	(E)-3-(3-(benzo[d][1,3]dioxol-5-yl)-1-phenyl-4,5-dihydro-1H-pyrazol-4-yl)-1-(2,4,6-trimethoxyphenyl)prop-2-en-1-one	$C_{28}H_{26}N_2O_6$
142	(E)-3-(3-(benzo[d][1,3]dioxol-5-yl)-1-phenyl-4,5-dihydro-1H-pyrazol-4-yl)-1-(2,3,4-trimethoxyphenyl)prop-2-en-1-one	$C_{28}H_{26}N_2O_6$
143	(E)-3-(3-(benzo[d][1,3]dioxol-5-yl)-1-phenyl-4,5-dihydro-1H-pyrazol-4-yl)-1-(3,4-dimethoxyphenyl)prop-2-en-1-one	$C_{27}H_{24}N_2O_5$
144	(E)-3-(3-(benzo[d][1,3]dioxol-5-yl)-1-phenyl-4,5-dihydro-1H-pyrazol-4-yl)-1-(2,5-dimethoxyphenyl)prop-2-en-1-one	$C_{27}H_{24}N_2O_5$

145	(E)-3-(3-(benzo[d][1,3]dioxol-5-yl)-1-phenyl-4,5-dihydro-1H-pyrazol-4-yl)-1-(2,4-dimethoxyphenyl)prop-2-en-1-one	$C_{27}H_{24}N_2O_5$
146	(E)-3-(3-(benzo[d][1,3]dioxol-5-yl)-1-phenyl-4,5-dihydro-1H-pyrazol-4-yl)-1-(thiophen-3-yl)prop-2-en-1-one	$C_{23}H_{18}N_2O_3S$
147	(E)-3-(3-(benzo[d][1,3]dioxol-5-yl)-1-phenyl-4,5-dihydro-1H-pyrazol-4-yl)-1-(pyridin-2-yl)prop-2-en-1-one	$C_{24}H_{19}N_3O_3$
148	(E)-3-(3-(benzo[d][1,3]dioxol-5-yl)-1-phenyl-4,5-dihydro-1H-pyrazol-4-yl)-1-(pyridin-3-yl)prop-2-en-1-one	$C_{24}H_{19}N_3O_3$
149	(E)-1-(benzo[d][1,3]dioxol-5-yl)-3-(3-(benzo[d][1,3]dioxol-5-yl)-1-phenyl-4,5-dihydro-1H-pyrazol-4-yl)prop-2-en-1-one	$C_{26}H_{20}N_2O_5$
150	(E)-3-(3-(benzo[d][1,3]dioxol-5-yl)-1-phenyl-4,5-dihydro-1H-pyrazol-4-yl)-1-(1H-pyrrol-3-yl)prop-2-en-1-one	$C_{23}H_{19}N_3O_3$
151	(E)-3-(1-phenyl-3-(pyridin-3-yl)-4,5-dihydro-1H-pyrazol-4-yl)-1-(thiophen-3-yl)prop-2-en-1-one	$C_{21}H_{17}N_3OS$
152	(E)-3-(1-phenyl-3-(pyridin-3-yl)-4,5-dihydro-1H-pyrazol-4-yl)-1-(pyridin-2-yl)prop-2-en-1-one	$C_{22}H_{18}N_4O$
153	(E)-1-(benzo[d][1,3]dioxol-5-yl)-3-(1-phenyl-3-(pyridin-3-yl)-4,5-dihydro-1H-pyrazol-4-yl)prop-2-en-1-one	$C_{24}H_{19}N_3O_3$
154	(E)-3-(1-phenyl-3-(pyridin-3-yl)-4,5-dihydro-1H-pyrazol-4-yl)-1-(2H-pyrrol-4-yl)prop-2-en-1-one	$C_{21}H_{18}N_4O$
155	(E)-1-(1H-indol-3-yl)-3-(1-phenyl-3-(pyridin-3-yl)-4,5-dihydro-1H-pyrazol-4-yl)prop-2-en-1-one	$C_{25}H_{20}N_4O$
156	(E)-1-phenyl-3-(1-phenyl-3-(pyridin-3-yl)-4,5-dihydro-1H-pyrazol-4-yl)prop-2-en-1-one	$C_{23}H_{19}N_3O$
157	(E)-3-(1-phenyl-3-(pyridin-3-yl)-4,5-dihydro-1H-pyrazol-4-yl)-1-(3,4,5-trimethoxyphenyl)prop-2-en-1-one	$C_{26}H_{25}N_3O_4$
158	(E)-3-(1-phenyl-3-(pyridin-3-yl)-4,5-dihydro-1H-pyrazol-4-yl)-1-(2,4,6-trimethoxyphenyl)prop-2-en-1-one	$C_{26}H_{25}N_3O_4$
159	(E)-3-(1-phenyl-3-(pyridin-3-yl)-4,5-dihydro-1H-pyrazol-4-yl)-1-(2,3,4-trimethoxyphenyl)prop-2-en-1-one	$C_{26}H_{25}N_3O_4$
160	(E)-1-(3,4-dimethoxyphenyl)-3-(1-phenyl-3-(pyridin-3-yl)-4,5-dihydro-1H-pyrazol-4-yl)prop-2-en-1-one	$C_{25}H_{23}N_3O_3$
161	(E)-1-(2,5-dimethoxyphenyl)-3-(1-phenyl-3-(pyridin-3-yl)-4,5-dihydro-1H-pyrazol-4-yl)prop-2-en-1-one	$C_{25}H_{23}N_3O_3$
162	(E)-1-(2,4-dimethoxyphenyl)-3-(1-phenyl-3-(pyridin-3-yl)-4,5-dihydro-1H-pyrazol-4-yl)prop-2-en-1-one	$C_{25}H_{23}N_3O_3$
163	(E)-3-(1-phenyl-3-(pyridin-4-yl)-4,5-dihydro-1H-pyrazol-4-yl)-1-(thiophen-3-yl)prop-2-en-1-one	$C_{21}H_{17}N_3OS$
164	(E)-1-(benzo[d][1,3]dioxol-5-yl)-3-(1-phenyl-3-(pyridin-4-yl)-4,5-dihydro-1H-pyrazol-4-yl)prop-2-en-1-one	$C_{24}H_{19}N_3O_3$
165	(E)-3-(1-phenyl-3-(pyridin-4-yl)-4,5-dihydro-1H-pyrazol-4-yl)-1-(1H-pyrrol-3-yl)prop-2-en-1-one	$C_{21}H_{18}N_4O$
166	(E)-1-phenyl-3-(1-phenyl-3-(pyridin-4-yl)-4,5-dihydro-1H-pyrazol-4-yl)prop-2-en-1-one	$C_{23}H_{19}N_3O$
167	(E)-3-(1-phenyl-3-(pyridin-4-yl)-4,5-dihydro-1H-pyrazol-4-yl)-1-(3,4,5-trimethoxyphenyl)prop-2-en-1-one	$C_{26}H_{25}N_3O_4$

168	(E)-3-(1-phenyl-3-(pyridin-4-yl)-4,5-dihydro-1H-pyrazol-4-yl)-1-(2,4,6-trimethoxyphenyl)prop-2-en-1-one	$C_{26}H_{25}N_3O_4$
169	(E)-3-(1-phenyl-3-(pyridin-4-yl)-4,5-dihydro-1H-pyrazol-4-yl)-1-(2,3,4-trimethoxyphenyl)prop-2-en-1-one	$C_{26}H_{25}N_3O_4$
170	(E)-1-(3,4-dimethoxyphenyl)-3-(1-phenyl-3-(pyridin-4-yl)-4,5-dihydro-1H-pyrazol-4-yl)prop-2-en-1-one	$C_{25}H_{23}N_3O_3$
171	(E)-1-(2,5-dimethoxyphenyl)-3-(1-phenyl-3-(pyridin-4-yl)-4,5-dihydro-1H-pyrazol-4-yl)prop-2-en-1-one	$C_{25}H_{23}N_3O_3$
172	(E)-1-(2,4-dimethoxyphenyl)-3-(1-phenyl-3-(pyridin-4-yl)-4,5-dihydro-1H-pyrazol-4-yl)prop-2-en-1-one	$C_{25}H_{23}N_3O_3$
173	(E)-3-phenyl-1-(4-((1-phenyl-1H-tetrazol-5-yl)oxy)phenyl)prop-2-en-1-one	$C_{22}H_{16}N_4O_2$
174	(E)-3-(3,4-dimethoxyphenyl)-1-(4-((1-phenyl-1H-tetrazol-5-yl)oxy)phenyl)prop-2-en-1-one	$C_{24}H_{20}N_4O_4$
175	(E)-1-(4-((1-phenyl-1H-tetrazol-5-yl)oxy)phenyl)-3-(3,4,5-trimethoxyphenyl)prop-2-en-1-one	$C_{25}H_{22}N_4O_5$
176	(E)-3-(4-chlorophenyl)-1-(4-((1-phenyl-1H-tetrazol-5-yl)oxy)phenyl)prop-2-en-1-one	$C_{22}H_{15}ClN_4O_2$
177	(E)-3-(2-bromophenyl)-1-(4-((1-phenyl-1H-tetrazol-5-yl)oxy)phenyl)prop-2-en-1-one	$C_{22}H_{15}BrN_4O_2$
178	(E)-3-(4-bromophenyl)-1-(4-((1-phenyl-1H-tetrazol-5-yl)oxy)phenyl)prop-2-en-1-one	$C_{22}H_{15}BrN_4O_2$
179	(E)-3-(4-fluorophenyl)-1-(4-((1-phenyl-1H-tetrazol-5-yl)oxy)phenyl)prop-2-en-1-one	$C_{22}H_{15}FN_4O_2$
180	(E)-3-(2-nitrophenyl)-1-(4-((1-phenyl-1H-tetrazol-5-yl)oxy)phenyl)prop-2-en-1-one	$C_{22}H_{15}N_5O_4$
181	(E)-3-(4-methoxyphenyl)-1-(5-methyl-2-(methylamino)thiazol-4-yl)prop-2-en-1-one	$C_{15}H_{16}N_2O_2S$
182	(E)-1-(5-methyl-2-(methylamino)thiazol-4-yl)-3-(p-tolyl)prop-2-en-1-one	$C_{15}H_{16}N_2OS$
183	(E)-1-(5-methyl-2-(methylamino)thiazol-4-yl)-3-phenylprop-2-en-1-one	$C_{14}H_{14}N_2OS$
184	(E)-3-(4-fluorophenyl)-1-(5-methyl-2-(methylamino)thiazol-4-yl)prop-2-en-1-one	$C_{14}H_{13}FN_2OS$
185	(E)-3-(4-chlorophenyl)-1-(5-methyl-2-(methylamino)thiazol-4-yl)prop-2-en-1-one	$C_{14}H_{13}ClN_2OS$
186	(E)-3-(2,4-dichlorophenyl)-1-(5-methyl-2-(methylamino)thiazol-4-yl)prop-2-en-1-one	$C_{14}H_{12}Cl_2N_2OS$
187	(E)-1-phenyl-3-(4-(3-(4-(3,4,5-trimethoxyphenyl)thiazol-2-yl)-2,3-dihydroimidazo[1,2-a]pyridin-2-yl)phenyl)prop-2-en-1-one	$C_{34}H_{29}N_3O_4S$
188	(E)-1-(3,4,5-trimethoxyphenyl)-3-(4-(3-(4-(3,4,5-trimethoxyphenyl)thiazol-2-yl)-2,3-dihydroimidazo[1,2-a]pyridin-2-yl)phenyl)prop-2-en-1-one	$C_{37}H_{35}N_3O_7S$

189	(E)-1-(3,5-dimethoxyphenyl)-3-(4-(3-(4-(3,4,5-trimethoxyphenyl)thiazol-2-yl)-2,3-dihydroimidazo[1,2-a]pyridin-2-yl)phenyl)prop-2-en-1-one	$C_{36}H_{33}N_3O_6S$
190	(E)-1-(4-methoxyphenyl)-3-(4-(3-(4-(3,4,5-trimethoxyphenyl)thiazol-2-yl)-2,3-dihydroimidazo[1,2-a]pyridin-2-yl)phenyl)prop-2-en-1-one	$C_{35}H_{31}N_3O_5S$
191	(E)-1-(4-chlorophenyl)-3-(4-(3-(4-(3,4,5-trimethoxyphenyl)thiazol-2-yl)-2,3-dihydroimidazo[1,2-a]pyridin-2-yl)phenyl)prop-2-en-1-one	$C_{34}H_{28}ClN_3O_4S$
192	(E)-1-(4-bromophenyl)-3-(4-(3-(4-(3,4,5-trimethoxyphenyl)thiazol-2-yl)-2,3-dihydroimidazo[1,2-a]pyridin-2-yl)phenyl)prop-2-en-1-one	$C_{34}H_{28}BrN_3O_4S$
193	(E)-1-(4-nitrophenyl)-3-(4-(3-(4-(3,4,5-trimethoxyphenyl)thiazol-2-yl)-2,3-dihydroimidazo[1,2-a]pyridin-2-yl)phenyl)prop-2-en-1-one	$C_{34}H_{28}N_4O_6S$
194	(E)-1-(3,5-dinitrophenyl)-3-(4-(3-(4-(3,4,5-trimethoxyphenyl)thiazol-2-yl)-2,3-dihydroimidazo[1,2-a]pyridin-2-yl)phenyl)prop-2-en-1-one	$C_{34}H_{27}N_5O_8S$
195	(E)-4-(3-(4-(3-(4-(3,4,5-trimethoxyphenyl)thiazol-2-yl)-2,3-dihydroimidazo[1,2-a]pyridin-2-yl)phenyl)acryloyl)benzonitrile	$C_{35}H_{28}N_4O_4S$
196	(E)-1-(p-tolyl)-3-(4-(3-(4-(3,4,5-trimethoxyphenyl)thiazol-2-yl)-2,3-dihydroimidazo[1,2-a]pyridin-2-yl)phenyl)prop-2-en-1-one	$C_{35}H_{31}N_3O_4S$
197	(E)-2-((4-allyl-5-(4-methoxyphenyl)-4H-1,2,4-triazol-3-yl)thio)-N-(4-(3-(3,4,5-trimethoxyphenyl)acryloyl)phenyl)acetamide	$C_{32}H_{32}N_4O_6S$
198	(E)-1-(3,4-dimethoxyphenyl)-3-(3-ethoxy-4-((1-(2-hydroxyphenyl)-1H-1,2,3-triazol-4-yl)methoxy)phenyl)prop-2-en-1-one	$C_{28}H_{27}N_3O_6$
199	(E)-1-(4-bromophenyl)-3-(3-ethoxy-4-((1-(2-hydroxyphenyl)-1H-1,2,3-triazol-4-yl)methoxy)phenyl)prop-2-en-1-one	$C_{26}H_{22}BrN_3O_4$
200	(E)-3-(3-ethoxy-4-((1-(2-hydroxyphenyl)-1H-1,2,3-triazol-4-yl)methoxy)phenyl)-1-(4-hydroxyphenyl)prop-2-en-1-one	$C_{26}H_{23}N_3O_5$
201	(E)-1-(3,4-dimethoxyphenyl)-3-(3-ethoxy-4-((1-(2-hydroxy-4-methylphenyl)-1H-1,2,3-triazol-4-yl)methoxy)phenyl)prop-2-en-1-one	$C_{29}H_{29}N_3O_6$
202	(E)-1-(4-bromophenyl)-3-(3-ethoxy-4-((1-(2-hydroxy-4-methylphenyl)-1H-1,2,3-triazol-4-yl)methoxy)phenyl)prop-2-en-1-one	$C_{27}H_{24}BrN_3O_4$
203	(E)-3-(3-ethoxy-4-((1-(2-hydroxy-4-methylphenyl)-1H-1,2,3-triazol-4-yl)methoxy)phenyl)-1-(3-hydroxyphenyl)prop-2-en-1-one	$C_{27}H_{25}N_3O_5$
204	(E)-3-(4-((1-(4-chloro-2-hydroxyphenyl)-1H-1,2,3-triazol-4-yl)methoxy)-3-ethoxyphenyl)-1-(3,4-dimethoxyphenyl)prop-2-en-1-one	$C_{28}H_{26}ClN_3O_6$
205	(E)-1-(4-bromophenyl)-3-(4-((1-(4-chloro-2-hydroxyphenyl)-1H-1,2,3-triazol-4-yl)methoxy)-3-ethoxyphenyl)prop-2-en-1-one	$C_{26}H_{21}BrClN_3O_4$
206	(E)-3-(4-((1-(4-chloro-2-hydroxyphenyl)-1H-1,2,3-triazol-4-yl)methoxy)-3-ethoxyphenyl)-1-(3-hydroxyphenyl)prop-2-en-1-one	$C_{26}H_{22}ClN_3O_5$
